# Influence of *Cryptosporidium* and rotavirus co-infection on infectivity in calves

**DOI:** 10.3389/fvets.2026.1715161

**Published:** 2026-02-17

**Authors:** Fumi Murakoshi, Megumi Itoh, Rofaida Mostafa Soliman, Tatsunori Masatani, Kenichi Shibano, Takaaki Nakaya, Kentaro Kato

**Affiliations:** 1 Laboratory of Veterinary Microbiology, Tokyo University of Agriculture and Technology, Tokyo, Japan; 2 Frontier Research Institute for Interdisciplinary Sciences, Tohoku University, Sendai, Japan; 3 Laboratory of Sustainable Animal Environment, Graduate School of Agricultural Science, Tohoku University, Osaki, Miyagi, Japan; 4 Department of Veterinary Medicine, Obihiro University of Agriculture and Veterinary Medicine, Obihiro, Hokkaido, Japan; 5 Department of Infectious Diseases, Kyoto Prefectural University of Medicine, Kyoto, Japan; 6 Department of Infectious Diseases and Epidemics, Faculty of Veterinary Medicine, Damanhour University, Damanhour, El-Beheira, Egypt; 7 Laboratory of Zoonotic Diseases, Faculty of Applied Biological Sciences, Gifu University, Gifu, Japan; 8 Center for One Medicine Innovative Translational Research (COMIT), Institute for Advanced Study, Gifu University, Gifu, Japan; 9 Faculty of Veterinary Medicine, Okayama University of Science, Okayama, Japan

**Keywords:** bovine rotavirus, cattle, co-infection, *Cryptosporidium parvum*, enteric infection

## Abstract

Rotavirus A (RVA; species *Rotavirus alphagastroenteritidis*) and *Cryptosporidium* spp. are major enteric pathogens in infants and neonatal calves, causing severe diarrhea that can lead to fatal outcomes. These pathogens thus pose challenges in both public health and the livestock industries. Although co-infections are common, their pathogenesis remains poorly understood. Here, we conducted a longitudinal investigation in naturally infected calves to assess the impact of co-infection with rotavirus and *Cryptosporidium*. Infection status was determined based on daily fecal antigen testing and oocyst per gram (OPG) counts from birth to 22 days of age. Based on these criteria, seven calves were classified as having *Cryptosporidium* mono-infection and three calves as having mixed infection. We found that subclinical infection with bovine rotavirus significantly shortened the duration of diarrhea caused by *Cryptosporidium parvum* in calves and reduced initial oocyst shedding. Furthermore, *in vitro* experiments using the bovine intestinal epitheliocyte (BIE) cell line demonstrated that the BRV Lincoln strain (G6, P[1]) non-structural protein 4 (NSP4) inhibits *C. parvum* infection, possibly by interfering with the host sodium-glucose co-transporter 1 (SGLT1). Our study highlights a potential novel strategy for controlling both BRV and *C. parvum* by exploiting their interactions during co-infection.

## 1 Introduction

Diarrheal diseases are a major cause of morbidity and mortality in both humans and livestock worldwide. In the livestock industry, neonatal diarrhea in calves leads to significant economic losses due to growth retardation, increased mortality, and reduced productivity (1–3). Among young ruminants, the main causative agents include *bovine rotavirus*, *Cryptosporidium parvum*, *Escherichia coli*, *Salmonella enterica*, and *Clostridium perfringens* (4). Notably, *C. parvum* and bovine rotavirus A are among the leading causes of diarrhea in neonatal calves (5).

*Cryptosporidium parvum* is a zoonotic protozoan parasite capable of infecting more than 150 vertebrate species, including humans (6). Upon oral ingestion, the parasite proliferates in the intestinal epithelium and releases oocysts that are highly resistant to chlorine-based disinfectants, facilitating environmental persistence. In calves, prophylactic administration of halofuginone lactate or lasalocid-NA has been shown to reduce the incidence of cryptosporidiosis (7, 8). Recently, passive immunization strategies, such as maternal vaccination using *C. parvum* antigen–based vaccines (e.g., Bovilis Cryptium^®^, MSD Animal Health), have also been developed to prevent neonatal infection (9).

Rotavirus is a genus of double-stranded RNA viruses within the family *Reoviridae* and comprises at least 11 recognized species (A–D, F–L). Among these, rotavirus A (species Rotavirus alphagastroenteritidis), commonly referred to as bovine rotavirus (BRV) in cattle, is a major enteric pathogen in neonatal calves. In cattle, an inactivated vaccine is available for use in pregnant cows to enhance colostral antibodies, thereby preventing neonatal rotavirus disease (10). However, no effective antiviral treatment is currently available.

Co-infections with rotavirus and *C. parvum* have been reported in calves and may result from overlapping susceptibility periods: *C. parvum* commonly infects calves aged 1–21 days (11), whereas BRV primarily affects those aged 1–14 days. Previous studies reported inconsistent findings regarding the clinical outcomes of co-infection (12, 13), and most lacked information on infection timing or prior exposure. Therefore, longitudinal investigations are necessary to clarify the pathophysiological interactions between these two pathogens in neonatal calves. Rotavirus non-structural protein 4 (NSP4) is a viral enterotoxin, and its biologically active regions have been identified in previous studies. Notably, the NSP4_114–135 peptide was originally described as a functional domain capable of inhibiting the Na^+^-D-glucose symporter (SGLT1) (14, 15).

In this study, we conducted a longitudinal investigation to assess the impact of co-infection with rotavirus and *C. parvum* in calves. In addition, we performed *in vitro* molecular analyses to elucidate the interactions between the two pathogens during co-infection.

## 2 Methods

### 2.1 Investigation of calves with mixed infections of BRV and *C. parvum*

#### 2.1.1 Information on the calves used in the experiment

From February to March 2016, fecal samples were collected daily from 10 calves at a dairy farm in Obihiro City, Hokkaido, Japan. This farm raises Holstein and Jersey cattle, calves were fed 4–6 L of colostrum within 24 h after birth. Insufficient colostrum intake in neonatal calves is associated with an increased risk of rotavirus-induced diarrhea. At the study farm, when the Brix value of colostrum was below 22% (approximately equivalent to an IgG concentration of 50 mg/mL), 450 g of a bovine colostrum powder supplement (Headstart^®^, Elanco, Indiana, U.S.) is administered. Rotavirus and *Cryptosporidium* infections have been recognized as major health issues in calves at this farm. No vaccines against either pathogen were used on this farm. In addition to the samples collected from this farm, fecal samples were included from two calves used in practical training at Obihiro University of Agriculture and Veterinary Medicine in December 2016. Twelve calves were initially enrolled, but data from eleven were analyzed after one was transferred to another farm. The calves included in this study ranged in age from 1 to 22 days and were sampled consecutively. The study population consisted of nine Holstein and two Jersey calves, all of which were clinically healthy at birth. Among the Jersey calves, one belonged to the co-infection group and the other to the *C. parvum*-infected group. Individual infection days for BRV and *C. parvum* for each calf are summarized in Supplementary Table S1. Because the primary objective of this field study was to evaluate the effect of prior rotavirus infection on subsequent *C. parvum*–associated disease, only calves in which rotavirus infection preceded *C. parvum* infection were included in the main comparative analyses. Of the 11 calves with complete longitudinal data, 7 calves with *C. parvum* mono-infection and 3 calves with rotavirus-preceding co-infection were included in the main comparative analyses.

#### 2.1.2 Fecal sample collection and pathogen detection

Housing conditions and fecal sample collection procedures were standardized throughout the study. All calves included in this study were housed individually in separate pens throughout the study period and were physically separated from other calves kept at the farm at the same time. This housing arrangement minimized the possibility of cross-contamination between animals. Fecal samples were collected daily directly from the rectum of each individual calf using disposable gloves. Fecal samples were collected in the morning, and their physical characteristics were recorded as diarrheic, soft, or normal. Diarrhea was defined as stools corresponding to types 6–7 on the Bristol Stool Scale (BSS). Subsequently, immunochromatographic test kits (DipFit Tetra Calf Scours; cat. no. BIO K 156, Bio-X Diagnostics S.A., Rochefort, Belgium) were used to detect infections caused by rotavirus, *Cryptosporidium*, coronavirus, and *Escherichia coli* (*E. coli*) K99 (F5). All tests were performed strictly according to the manufacturer’s instructions, without any modifications to the protocol. The collected fecal samples were stored at 4 °C. Each calf was monitored from birth (day 0) to day 22.

Shedding of *Cryptosporidium* oocysts was quantified by determining oocysts per gram (OPG) of feces using a standard sucrose flotation method, as described previously (16, 17). Briefly, one gram of feces was placed into a 15-mL tube, and 14 mL of sucrose solution (density 1.2 g/mL) was added. The mixture was centrifuged at 1,300 rpm (corresponding to approximately 300 × g, depending on rotor radius) for 10 min. After centrifugation, the tube was filled with sucrose solution, and a coverslip was gently placed on top. The preparation was allowed to stand for 30 min, after which *Cryptosporidium* oocysts adhering to the coverslip were counted under a light microscope.

#### 2.1.3 Species identification and subtyping using PCR

Genomic DNA was extracted from fecal samples (0.3–0.4 g) using the QIAamp Fast DNA Stool Mini Kit (cat. no. 51604; QIAGEN, Hilden, Germany), strictly following the manufacturer’s instructions. *Cryptosporidium* spp. were detected and subtyped by nested PCR amplification targeting a ~ 830 bp and a ~ 850 bp fragment of the small subunit (SSU) rRNA and 60-kDa glycoprotein (GP60) genes, respectively, as described previously (18, 19).

### 2.2 Culture of BRV and *Cryptosporidium parvum*

As host cells for BRV growth, African green monkey kidney cells (MA104 cells, RCB0994) were purchased from RIKEN BioResource Research Center and were maintained in minimum essential medium (MEM) (cat. no. 21443–15; Nacalai Tesque, Kyoto, Japan) supplemented with 10% fetal bovine serum (FBS), 100 U/mL penicillin, and 100 μg/mL streptomycin (cat. no. 09367–34; Nacalai Tesque, Kyoto, Japan). The bovine intestinal epitheliocyte (BIE) cell line (Passage 39) kindly provided by Dr. Hisashi Aso (Graduate school of agricultural science, Tohoku University, Japan) (20, 21) was used for BRV and *C. parvum* inoculation studies; BIE cells were cultured in DMEM medium (cat. no. 16971–55; Nacalai Tesque, Kyoto, Japan) with 10% FBS and 100 U/mL penicillin, and 100 μg/mL streptomycin. BIE cells reproduce key morphological and functional features of bovine intestinal epithelium, including microvilli formation, expression of tight-junction proteins, and the epithelial marker cytokeratin. They also retain the ability to differentiate into M-like cells under appropriate conditions, indicating preservation of epithelial characteristics (20, 21). Although it remains unclear whether BIE cells elicit interferon responses identical to those observed *in vivo* in bovine intestinal cells, their response pattern to rotavirus infection is comparable to that reported in other established *in vitro* rotavirus infection systems (22). Bovine rotavirus (BRV Lincoln strain (G6, P[1])) kindly provided by Dr. Kunitoshi Imai (Graduate School of Animal and Veterinary Sciences and Agriculture, Obihiro University of Agriculture and Veterinary Medicine, Japan) was propagated in the MA104 cell line (Passage 3) as previously described (23). Viral titers were determined using fluorescence focus units (FFU) as previously described (24). All virus stocks were stored at −80 °C until use. BRVs were activated by incubation with trypsin (final concentration 1 μg/mL; Sigma Aldrich, MO, USA) at 37 °C for 30 min prior to infection (25).

*Cryptosporidium parvum* oocysts, strain HNJ-1 (26, 27), were kindly provided by Dr. Makoto Matsubayashi (Graduate school of veterinary science, Osaka Prefecture University, Japan). Oocysts were maintained by passage in experimentally infected SCID mice (C.B-17/Icr-*scid*/*scid*Jcl) (CLEA Japan, Inc., Tokyo, Japan) and were purified from feces by using discontinuous sucrose and cesium chloride gradients as described previously (17). *C. parvum* oocysts less than 6 months since harvest were used in all experiments. Animal experiments were approved by the Ethical Committee of the Committee on Animal Experiments of the Kyoto Prefectural University of Medicine (M2019-226, M2020-247, M2021-272).

### 2.3 Co-infection of BIE cells with BRV and *C. parvum*

*Cryptosporidium parvum* oocysts were bleached with 10% (v/v) purelox (OyaloxCo.Ltd., Tokyo, Japan) on ice for 15 min, then washed three times with ice-cold phosphate-buffered saline (PBS) and incubated with 0.2 mM sodium taurocholate (Nacalai Tesque, Kyoto, Japan) at 37 °C for 30 min to stimulate excystation. BIE cells (5 × 10^4^ cells/well) were seeded in 96-well plates and cultured for 24 h. BRV (100 μL) was incubated with trypsin at a final concentration of 1 μg/mL at 37 °C for 30 min. Following incubation, 900 μL of serum-free DMEM (cat. no. 16971–55; Nacalai Tesque, Kyoto, Japan) containing 1 μg/mL trypsin was added to the BRV-containing tube, and the resulting suspension was inoculated into 96-well plates at 100 μL per well (BRV, MOI = 1). Twelve hours after inoculation, *C. parvum* was excysted into sporozoites and added at a concentration of 5 × 10^4^ oocysts/100 μL (MOI = 1). The plate was incubated at 37 °C for 1.5 h, washed with serum-free DMEM containing trypsin, and cultured until 24 h post-rotavirus inoculation. The experiment was conducted using a rotavirus inoculum corresponding to an MOI of 1, as determined based on infectivity titration in MA104 cells, which was chosen to ensure robust infection while minimizing excessive cytopathic effects.

The cells were fixed and permeabilized with ice-cold 100% methanol (cat. no. 137–01823; FUJIFILM Wako Pure Chemical Corporation, Osaka, Japan) for 10 min and subsequently washed three times with PBS. Blocking was performed with 1% BSA (cat. no. 017–15,124, FUJIFILM Wako Pure Chemical Corporation, Osaka, Japan) in PBS for 30 min. The cells were then stained for 1 h with Sporo-Glo, an anti*-Cryptosporidium* polyclonal antibody (cat. no. A600Cy3-R-1X; Waterborne Environmental, Inc., Virginia, USA), according to the manufacturer’s instructions.

Infection with BRV induced a mild cytopathic effect (CPE) in BIE cells, characterized by partial cell detachment and a reduction in cell density. Quantitative image analysis revealed that the total number of adherent cells in BRV-infected wells was reduced by approximately 10–20% compared with non-infected control wells at 24 h post-inoculation. Because this reduction in viable cell numbers could affect the apparent frequency of subsequent *C. parvum* infection, the number of *C. parvum*-infected cells was normalized to the total number of viable cells in each well. Viable cell numbers were quantified using an IN Cell Analyzer 2,200 (GE Healthcare, Illinois, USA), with 42 fields acquired per well using a 20 × objective lens. The number of visible cell nuclei was determined using IN Cell Developer Toolbox software (GE Healthcare, Illinois, USA), and *C. parvum* infection rates were calculated as the proportion of infected cells relative to the total number of viable cells. BRV infection was confirmed by immunofluorescence assay (IFA). The cells were fixed with ice-cold 100% methanol for 10 min. Goat anti-BRV polyclonal antibodies (cat. no. ab20036; Abcam, Cambridge, UK) were used as primary antibodies to detect BRV antigens. Donkey anti-goat IgG conjugated with Alexa Fluor 555 (cat. no. AP180C; Thermo Fisher Scientific, MA, USA) was used as the secondary antibody. Images were captured using a Keyence BZ-X810 microscope (KEYENCE, Osaka, Japan). *Cryptosporidium* parasites were counted in 15 random fields per well using a 20 × objective lens.

### 2.4 Infection of poly(I:C)-transfected cells with *C. parvum*

Poly(I:C) (a synthetic double-stranded RNA (dsRNA)) (42,424, Tocris Bioscience, Bristol, UK) molecule (50 ng) was transfected into BIE cells seeded in a 96-well plate by using the PEI MAX (Polysciences, Inc., PA, USA), according to the manufacturer’s instruction. Transfection of BIE cells with poly(I:C) prior to *C. parvum* infection was performed to induce the cellular response to dsRNA before infection. Six hours after the transfection, the BIE cells were infected with *C. parvum* (MOI = 1). Three hours post-infection, the cells were washed. The number of *C. parvum-*infected BIE cells was counted 24 h after transfection. In a separate well, a similar experiment was conducted, and RNA was extracted from the BIE cells 24 h after transfection. Total RNA was extracted from each sample using the SV Total RNA Isolation System (Promega, WI, USA); reverse transcription was performed using the ReverTra Ace qPCR RT Master Mix (TOYOBO, Osaka, Japan). Interferon β expression was confirmed by PCR using the following primers: BtIFN-βF (5′-CTTTCCAGGAGCTACAGCTTGC-3′) and BtIFNβ-R (5′-ACGACTGTCCAGGCACACCTG-3′). KOD FX Neo (TOYOBO, Japan) was used for PCR amplification; the amplicon length was 435 bp.

### 2.5 Experimental inhibition of BIE cell infection with *C. parvum* by NSP4 peptide

We commissioned GenScript to synthesize the following peptides: NSP4_114–135: DKLTTREIEQVELLKRIYDKLT, and scrambled peptide: IDTKLDLLYRKRKIQLVETETE. The scrambled peptide had a completely random amino acid sequence. Cytotoxicity was evaluated using the 3-(4,5-dimethylthiazol-2-yl)-2,5-diphenyltetrazolium bromide (MTT) assay with the Cell Count Reagent SF (Nacalai Tesque, Kyoto, Japan). Absorbance was measured at 450 nm using a TriStar LB 941 microplate reader (Berthold Technologies, Bad Wildbad, Germany). BIE cells were seeded in 96-well plates at a density of 5 × 10^4^ cells/well and allowed to grow overnight. As previously described, *C. parvum* oocysts were excysted into sporozoites immediately before infection. BIE cells were then infected with *C. parvum* (MOI = 1) and treated separately with one of the following reagents in DMEM medium: 50 μM NSP4 peptide, 50 μM scrambled peptide, 0.05% phloridzin n-hydrate (approximately 0.3 μM; FUJIFILM Wako Pure Chemical Corporation, Osaka, Japan), or 10 μM nitazoxanide (TCI, Tokyo, Japan), which served as a positive control. Each condition was tested in triplicate wells, and the experiment was independently repeated three times. Three hours after infection, the cells were washed once. The peptide or drug (phloridzin or nitazoxanide) at the same concentration as used above was added to the medium after washing. Twenty-four hours after infection, the number of *C. parvum* infections was counted using the method described previously.

The cytotoxicity of the NSP4_114–135 peptide and scrambled peptide was tested across concentrations ranging from 30 to 200 μg/mL, and no significant toxicity was observed within this range. Based on these results, a concentration of 50 μg/mL was selected for subsequent experiments. Phlorizin was employed as a specific inhibitor of SGLT1, and nitazoxanide served as a positive control.

### 2.6 Experimental inhibition of glucose uptake in BIE cells

BIE cells seeded in a 96-well plate were infected with *C. parvum* as described above. At 50 min post-infection, 2-NBDG [2-(N-(7-Nitrobenz-2-oxa-1,3-diazol-4-yl)Amino) (Cayman Chemical, Michigan, USA)] was added to achieve a final concentration of 200 μM. After a 30-min incubation, the cells were washed twice with 1 × PBS. Because 2NBDG is a fluorescent analog of glucose, it was used to examine whether glucose uptake is increased at sites of *C. parvum* infection. The fluorescence of 2-NBDG was then observed under a fluorescence microscope at 200 × magnification. Fluorescent areas were counted in 10 random fields of view to assess glucose uptake.

### 2.7 Statistical analyses

**Figure 1:**
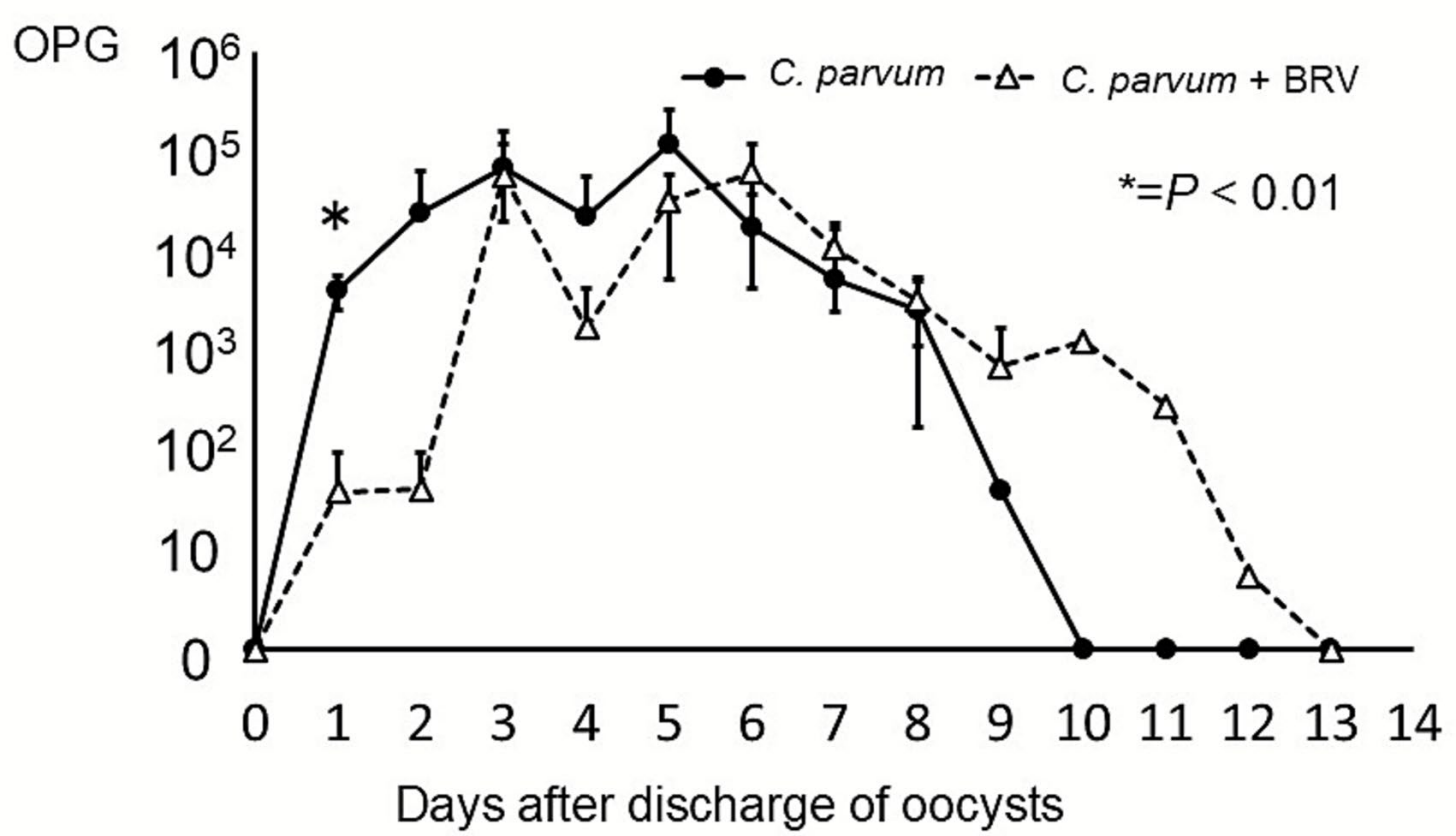
Oocyst shedding levels in calves infected with *C. parvum* only and calves co-infected with BRV and *C. parvum*. The solid line represents the oocyst shedding levels of *C. parvum*-infected calves, while the dashed line represents the oocyst shedding of calves co-infected with *C. parvum* and BRV. *p < 0.01.

**Figure 2:**
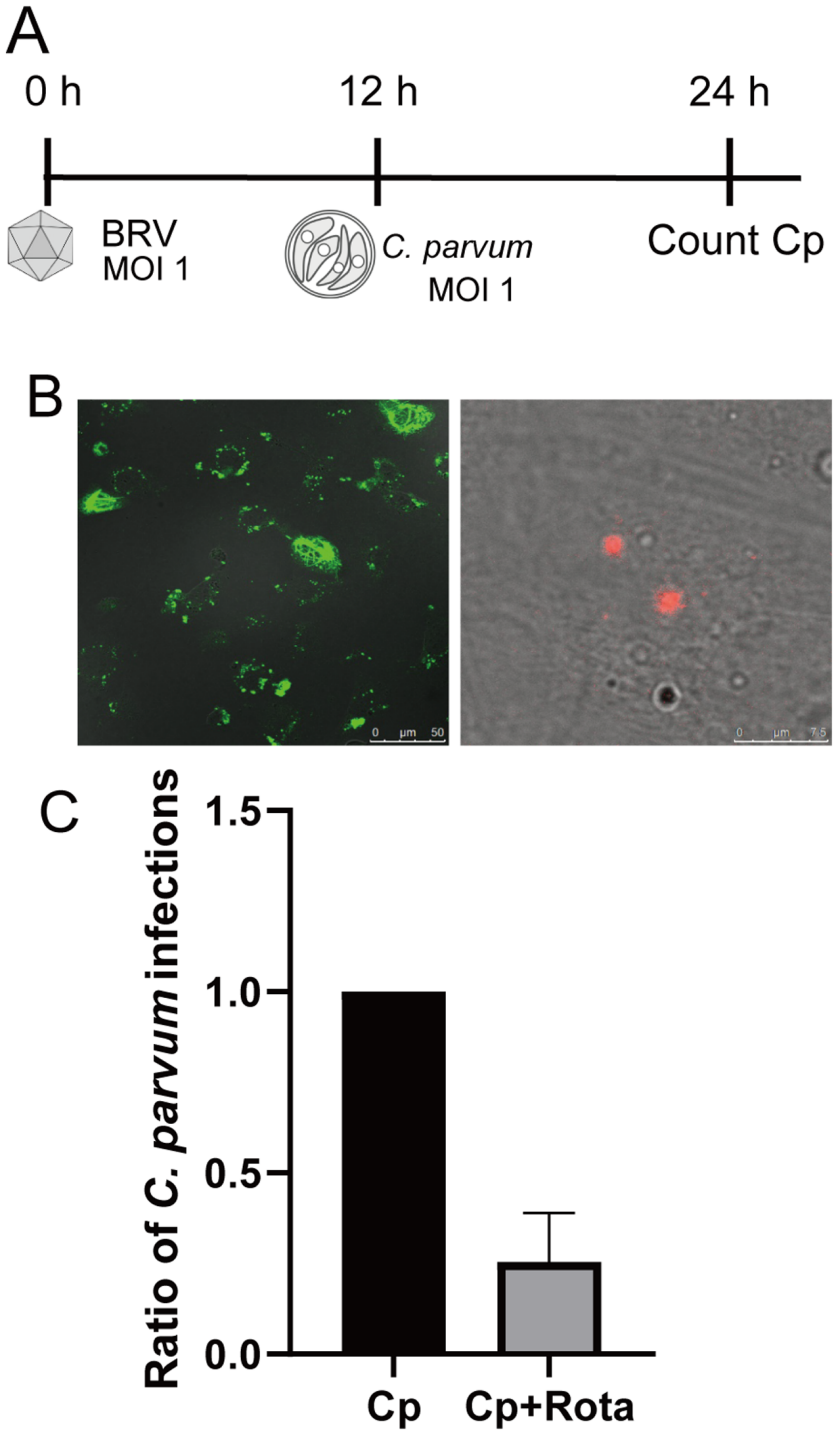
Co-infection of BIE cells with BRV and *C. parvum*. **(A)** BIE cells were infected with BRV (MOI = 1) for 12 and then challenged with *C. parvum* (MOI = 1). Intracellular *C. parvum* was quantified at 24 h post-rotavirus infection. **(B)** Immunofluorescence staining of BIE cells co-infected with BRV and *C. parvum*. The left panel shows BRV; the right panel shows *C. parvum*. **(C)** Comparison of the number of *C. parvum* infections in cells infected with *C. parvum* alone versus those co-infected with BRV. The number of *C. parvum* infections was normalized to the number of viable cells. *p < 0.05.

**Figure 3:**
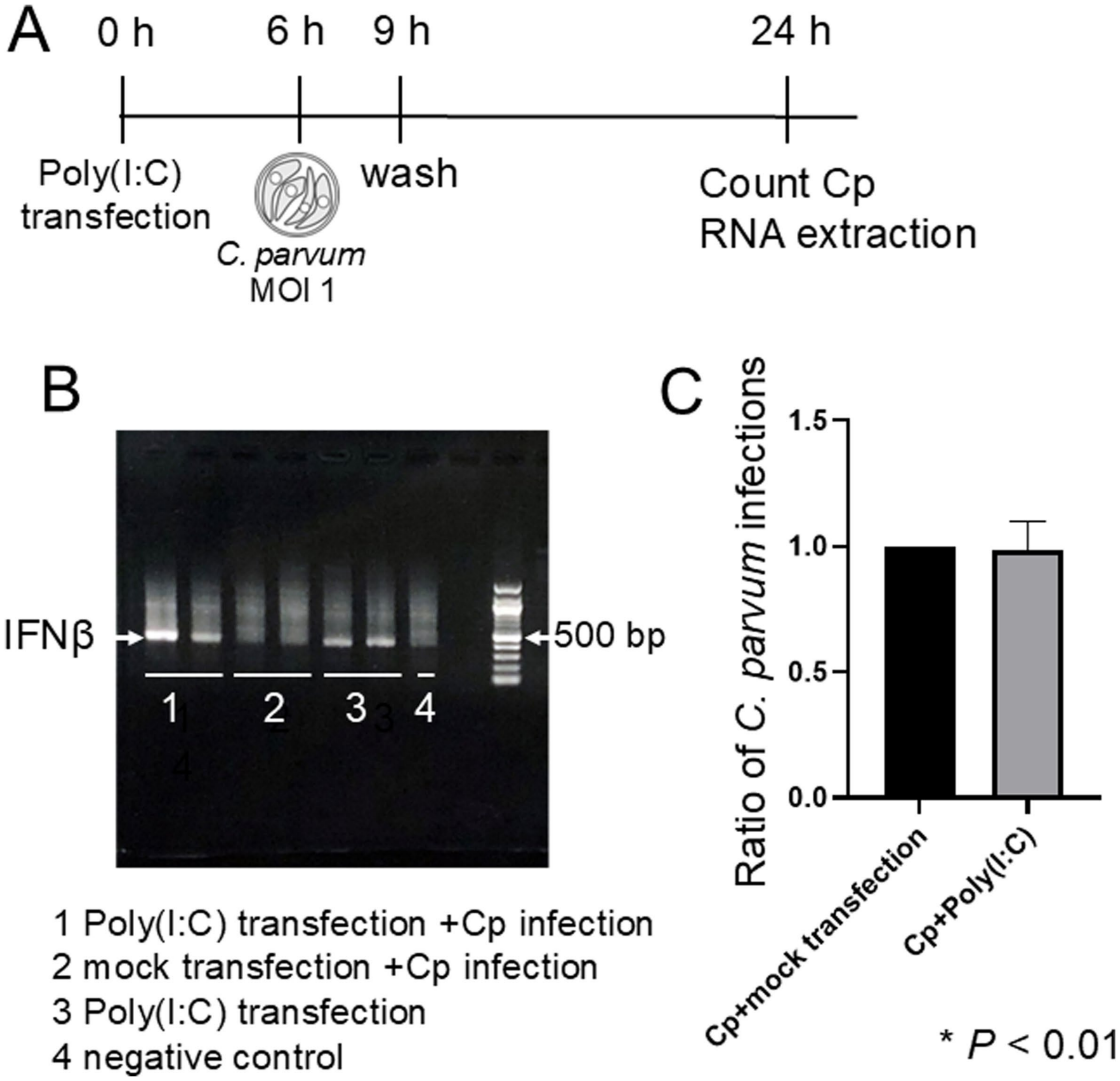
IFN-β induction by poly(I:C) transfection does not affect *C. parvum* infection. **(A)** Experimental design for poly(I:C) transfection and *C. parvum* infection. BIE cells were transfected with poly(I:C), and 6 h post-transfection, the cells were inoculated with *C. parvum*. The number of *C. parvum* infections was counted 24 h after transfection. **(B)** Total RNA was extracted from poly(I:C)-transfected cells, and RT-PCR was performed to detect IFN-β expression. Gel electrophoresis confirmed the presence of the IFN-β band (indicated by the arrow at approximately 435 bp). The IFN-β band was observed only in cells transfected with poly(I:C). Groups: 1, poly(I:C) + *C. parvum*; 2, mock transfection + *C. parvum*; 3, poly (I:C) alone; 4, negative control. **(C)** Comparison of *C. parvum* infection rates in cells transfected with poly (I:C) versus mock-transfected cells. No significant difference was observed.

**Figure 4:**
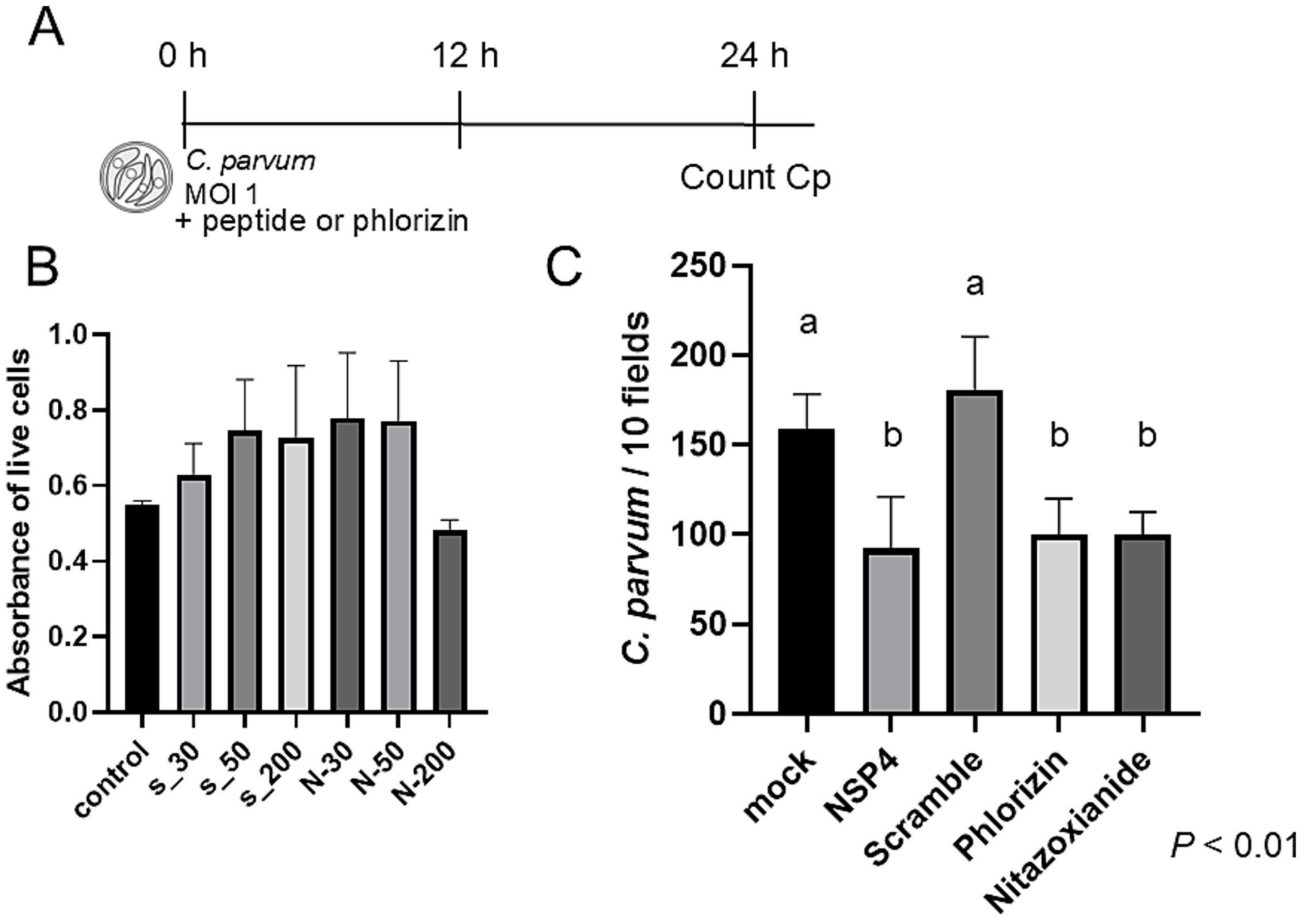
NSP4 peptide inhibits *C. parvum* infection. **(A)** BIE cells were infected with *C. parvum* (MOI = 1) in the presence or absence of NSP4_114–135_ peptide or scrambled control peptide. **(B)** The cytotoxicity of scrambled peptide (S-) and NSP4 peptide (N-) at concentrations ranging from 30 μg/mL to 200 μg/mL was evaluated using an MTT assay. No significant differences compared to the control were observed at any concentration. Consequently, a concentration of 50 μg/mL was used in subsequent experiments. **(C)** The number of *C. parvum* infections significantly decreased in cells treated with NSP4 peptide or phlorizin. Phlorizin is a specific inhibitor of SGLT1; nitazoxanide, an agent with limited therapeutic effect against *C. parvum*, served as a positive control. Significant differences were observed between groups with different letters (*p* < 0.01).

**Figure 5:**
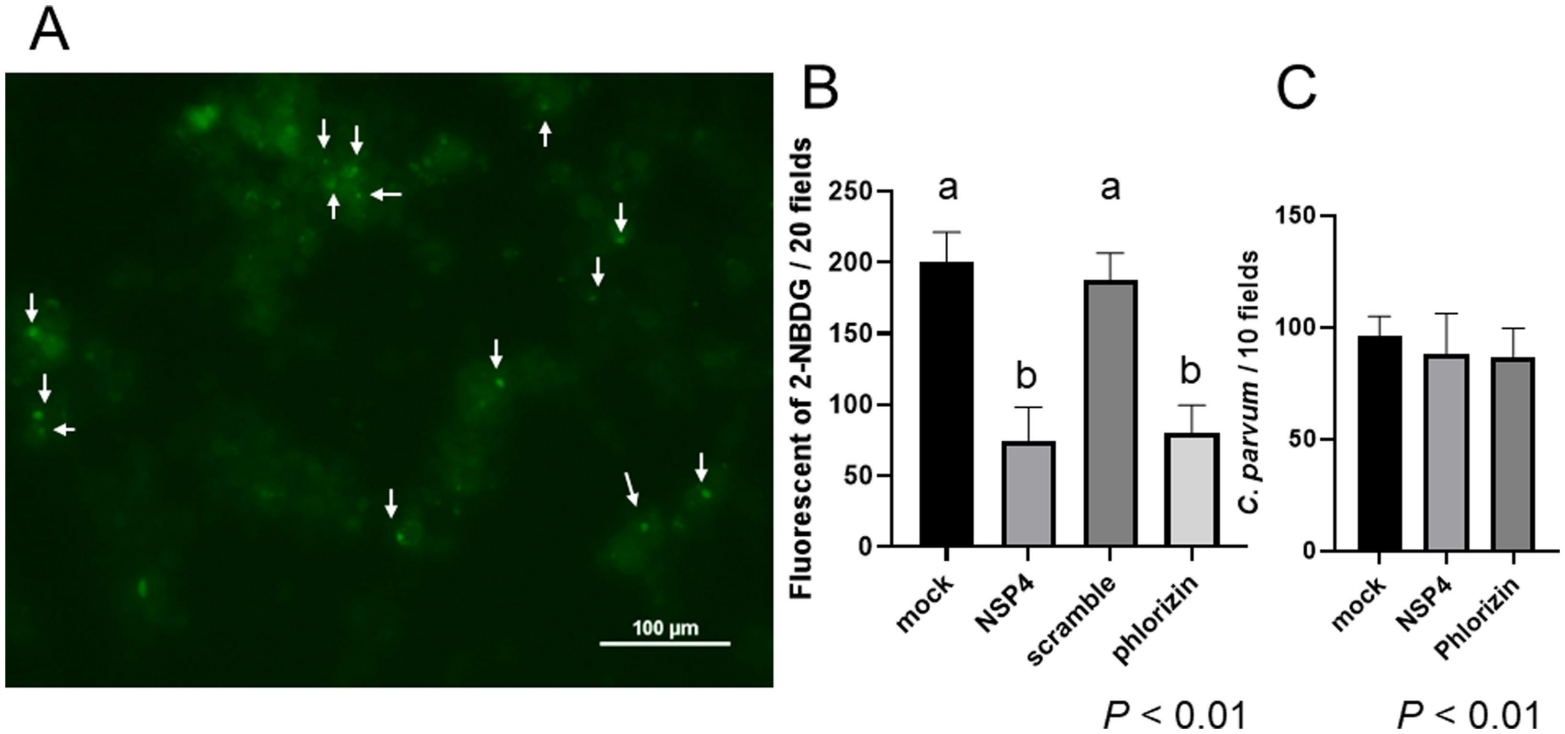
NSP4 peptide inhibits glucose uptake at *C. parvum* infection sites but cannot block initial invasion. BIE cells were infected with *C. parvum*, and 50 min post-infection, 2-NBDG (a fluorescent glucose analog) and either phlorizin (a specific SGLT1 inhibitor) or NSP4 peptide were added. The uptake of 2-NBDG was measured 30 min after addition. **(A)** Dot-like fluorescence of 2-NBDG (indicated by arrows) was observed at potential sites of *C. parvum* infection. **(B)** In *C. parvum*-infected BIE cells, the number of 2-NBDG-positive dots was significantly reduced in the NSP4 peptide and phlorizin-treated groups. Significant differences were observed between groups with different letters (*p* < 0.01). **(C)** BIE cells were treated with NSP4 peptide or phlorizin and then infected with *C. parvum*. The number of *C. parvum* infections was assessed 3 h post-infection. No significant change in number of infections was observed.

For *in vitro* experiments, three technical replicates were performed per experiment and the average value was determined. The experimental points represent an average of each three biological replicates (three independent experiments). Statistical analyses were performed using Prism7 (GraphPad Software, MA, USA). Statistical analyses were performed as follows: Mann–Whitney U test was used to compare the duration of diarrhea and oocyst shedding Table 1, and oocyst output was analyzed using two-way ANOVA followed by multiple comparisons (Figure 1). Data shown in Figures 2, 3 were analyzed using an unpaired *t*-test, while Figures 4, 5 were analyzed using one-way ANOVA. Differences were considered statistically significant at *p* < 0.05. Because of the small sample size in the field study (*n* = 7 and *n* = 3) and the uncertainty regarding the normality of the data distribution, a non-parametric Mann–Whitney U test was used for comparisons of diarrhea duration and initial oocyst shedding between mono-infected and co-infected calves. To further evaluate the robustness of the results, Welch’s *t*-test, which is more tolerant of unequal variances and sample sizes than Student’s t-test, was also performed, yielding the same qualitative conclusion. Therefore, the observed statistical significance was not dependent on the choice of parametric versus non-parametric testing.

**Table 1:** Oocyst shedding period and diarrhea duration in calves with *C. parvum* infection and calves co-infected with BRV and *C. parvum.*

Group	Oocyst discharge period (days)	Duration of diarrhea (days)
*C. parvum* infection (*N* = 7)	7.4*	4.4*
Mixed infection (*N* = 3)	9.0*	1.3*

*p < 0.04.

## 3 Results

### 3.1 Calves co-infected with BRV and *C. parvum* exhibit a significantly shorter duration of diarrhea and a reduction in initial oocyst shedding

All 11 analyzed calves were infected with *Cryptosporidium* within the first 16 days of life. Of these, four exhibited mixed infections with BRV and *Cryptosporidium* (Calves A–D; see Supplementary Table S1). Immunochromatographic test results were negative for coronavirus and *Escherichia coli*. All *Cryptosporidium* isolates from the calves were identified as *C. parvum*. Subtyping revealed that one calf (Calf C) had a mixed infection with subtypes IIaA15G2R1 and IIaA16G2R1, whereas the remaining calves were all infected with subtype IIaA16G3R1. Of the four co-infected calves, three acquired rotavirus before *C. parvum*, whereas one calf (Calf D) showed the reverse order. Therefore, because the aim was to assess the effect of prior rotavirus infection on subsequent *C. parvum*–associated disease, Calf D was excluded from the main comparative analyses. Consequently, the present analyses focused on seven calves with *C. parvum* mono-infection and only three calves that acquired *C. parvum* following rotavirus infection. The clinical course of Calf D is shown in Supplementary Table S1. This calf exhibited a diarrhea duration and oocyst shedding pattern comparable to those observed in calves with *C. parvum* mono-infection.

Table 1 shows the duration of oocyst shedding and the number of days calves experienced watery diarrhea. Diarrhea lasted significantly shorter in co-infected calves, averaging 1 day. In contrast, calves with *C. parvum* infection alone typically developed severe diarrhea concurrently with or the day after the onset of oocyst shedding, with a mean diarrhea duration of 4.4 days. However, the co-infected calves had a significantly longer duration of oocyst shedding. Figure 1 shows the oocyst shedding patterns. On the first day of shedding, the OPG in co-infected calves was significantly lower than that in singly infected calves. The total oocyst output was not statistically significant. Interestingly, in the co-infected calves, no symptoms of diarrhea were observed during the initial rotavirus infection, indicating that a subclinical infection had occurred (Supplementary Table S1). This supports that rotavirus infection occurred under conditions of sufficient maternal immunity.

### 3.2 Co-infection of BIE cells with BRV and *Cryptosporidium parvum* results in a reduction in the number of *C. parvum* infections

To investigate the initial response during co-infection with BRV and *C. parvum*, we examined changes in the number of *C. parvum* infections in BIE cells (Figures 2A, B). The results revealed a significant reduction in the number of *C. parvum* infections in the group co-infected with BRV (Figure 2C).

### 3.3 The reduction in *C. parvum* infections during co-infection is unrelated to the type I interferon response induced by viral infection

To investigate the effect of type I interferon responses induced by viral infection on *C. parvum* infection in more detail, BIE cells were transfected with poly(I:C), a synthetic analog of double-stranded RNA, to strongly induce type I interferon expression (Figure 3A). Upon poly(I:C) transfection, an IFN-β band was detected; however, no IFN-β band was observed in the group that was infected with *C. parvum* without poly(I:C) transfection (Figure 3B). Consequently, even when type I interferon expression was strongly induced by transfecting poly(I:C) into BIE cells, there were no significant changes in the number of *C. parvum* infections (Figure 3C). These findings suggest that the reduction in *C. parvum* infections during co-infection might involve factors beyond the innate immune response of cells.

### 3.4 NSP4 peptide-mediated inhibition of *C. parvum* infection in BIE cells

We next examined whether a rotavirus-derived peptide could directly affect *C. parvum* infection. BIE cells were treated with the NSP4_114–135 peptide during *C. parvum* infection (Figure 4A). A cytotoxicity assay confirmed that the NSP4_114–135 peptide was non-toxic at the concentration used (50 μg/mL) (Figure 4B). Treatment with the NSP4 peptide significantly reduced the number of *C. parvum*-infected cells compared with the scrambled peptide control. Similar inhibitory effects were observed with phlorizin, whereas nitazoxanide served as a positive control (Figure 4C).

### 3.5 NSP4 peptide inhibits the uptake of fluorescent glucose analogs at *C. parvum* infection sites

Since *C. parvum* infection requires SGLT1, localized glucose uptake is thought to occur at infection sites. To investigate this, a fluorescent glucose analog was added to determine whether its uptake was inhibited in the presence of the NSP4 peptide (Figure 5A). The number of punctate fluorescent signals of 2-NBDG was reduced in the groups treated with the NSP4 peptide or the SGLT1 inhibitor phlorizin during *C. parvum* infection (Figure 5B). These findings suggest that the NSP4 peptide inhibits the function of SGLT1 (sodium–glucose co-transport), thereby impairing the ability of *C. parvum* to achieve complete invasion of host cells. However, no significant changes in the number of *C. parvum* infections were observed at 3 h post-inoculation (Figure 5C), indicating that the inhibitory effect of NSP4 on *C. parvum* infection likely occurs more than 3 h post-inoculation.

## 4 Discussion

The prepatent period of BRV is 1–2 days, whereas that of *C. parvum* is 3–6 days (28, 29). Therefore, in farms where both pathogens are prevalent, co-infection typically occurs in the order of BRV followed by *C. parvum*. In this study, calves with such co-infections exhibited a significantly shorter duration of diarrhea.

A major limitation of the present field study is the very small number of calves in the co-infection group (*n* = 3). This reflects the constraints of working with naturally occurring infections, where both the timing and the sequence of pathogen exposure cannot be experimentally controlled. Consequently, although the observed shortening of diarrhea duration in calves that acquired *C. parvum* after rotavirus infection was statistically significant, this result should be interpreted with caution and should not be overgeneralized. The present data should be viewed as hypothesis-generating, providing preliminary evidence that rotavirus infection may modulate subsequent *C. parvum*-associated disease, rather than as a definitive demonstration of this effect. Larger, independent field studies will be required to confirm the robustness and generality of this finding.

Typically, rotavirus infection causes shortening, partial detachment, and destruction of intestinal villi, leading to diarrhea (30). However, in the present study, the calves in the co-infected group exhibited subclinical rotavirus infection, which suggests that colostrum intake was sufficient. Although the number of cases was limited to a single calf, one calf tested positive for *C. parvum* oocysts before testing positive for BRV; the duration of diarrhea in this calf was comparable to that observed in calves with mono-infections (Supplementary Table S1). The timing of infection likely affects disease severity (31, 32). The reduced oocyst shedding and diarrhea observed in co-infected calves may be associated with modulation of mucosal immune responses and barrier function. *C. parvum* infection is primarily controlled by IFN-*γ*–dependent Th1 responses, while IL-10 plays a protective role in preventing excessive intestinal inflammation. Rotavirus infection can transiently stimulate epithelial interferon signaling, particularly type III IFNs, and alter epithelial permeability through the action of NSP4 on tight junctions and sodium–glucose transporters. Rotavirus infection has been shown to increase IFN-γ and IL-10 levels in human cases of acute gastroenteritis (33) and to upregulate their expression *in vitro* using infected intestinal cell models (34). Such transient epithelial and immune activation might restrict *C. parvum* invasion and replication, while IL-10–mediated regulation could contribute to faster recovery of epithelial integrity and reduced diarrhea. Neonatal calves and human infants share several physiological characteristics that may influence susceptibility to enteric co-infections, including an immature intestinal barrier and developing mucosal immune system. However, differences in maternal antibody acquisition, environmental exposure, and microbial colonization patterns likely result in distinct infection dynamics between calves and human infants. Although these differences limit direct extrapolation, the epithelial and immune interactions identified in calves may provide useful insights into the mechanisms underlying co-infection in early human life.

Although serial quantification of both pathogens by qPCR would further strengthen the analyses of infection dynamics, this approach is technically difficult under field conditions, particularly when working with fecal samples that vary greatly in consistency and RNA recovery. In this study, *C. parvum* shedding was quantified by oocyst counts (OPG), which provide a reliable estimate of parasite burden, and rotavirus infection was monitored using an immunochromatographic test kit, which reflects the period of viral shedding in calves. We therefore consider the combination of OPG data and immunochromatographic detection to provide a reasonable approximation of infection timing and duration for both pathogens.

The observed reduction in diarrhea duration among co-infected calves should nevertheless be interpreted with caution, as factors such as age, colostrum intake, and immune status may also influence disease outcomes (35, 36). Although detailed immunological parameters were not available for all animals, all calves received colostrum and no signs of failure of passive transfer were recorded. While these factors may contribute to some variability in clinical response, they are unlikely to fully account for the marked difference in diarrhea duration. Future studies incorporating these parameters as covariates will be valuable to further clarify the relationship between co-infection and disease severity.

A study investigating the clinical significance of pathogen combinations in acute diarrhea among children in Rwanda and Zanzibar found that while certain pathogen combinations exacerbated symptoms, the combination of rotavirus and *Cryptosporidium* did not, supporting the findings of the present study (37). However, the mechanism underlying the significantly prolonged shedding of *C. parvum* oocysts in co-infected calves remains unclear. One possibility is that the reduction in the initial infection burden results in an inadequate immune response against *C. parvum*, leading to delayed clearance. The reduction in early *C. parvum* infection in calves may be attributed to cytokine responses induced by rotavirus infection. Supporting this hypothesis, IFN-*γ* has been reported to inhibit *C. parvum* infection (38). Thus, it is plausible that *in vivo*, BRV infection induces IFN-γ production in calves, thereby suppressing *C. parvum* infection. Further detailed studies of the immune response of calves to co-infection are needed. Since the decrease in cell count caused by BRV infection was normalized, the observed reduction in *C. parvum* infection cannot be explained solely by reduced cell availability, indicating that additional BRV-induced factors are likely involved. Previous studies suggest that the cytokines produced by the host *in vivo* influence the defense against *C. parvum* infection (39). However, our findings demonstrate that *in vitro*, BRV infection inhibits *C. parvum* infection via a mechanism independent of type I and type II IFNs. To explore a potential epithelial-level mechanism underlying this IFN-independent inhibition, we focused on rotavirus enterotoxin with a well-characterized biologically active region.

We identified the NSP4_114–135 peptide of BRV as a potential inhibitory factor against *C. parvum* infection. Because this peptide has been reported to act as a non-competitive and specific inhibitor of the Na^+^-D-glucose symporter (SGLT1) (14), is consistent with a mechanism that could account for the reduced *C. parvum* infection observed in our *in vitro* experiments. NSP4 is the only rotavirus protein known to function as an enterotoxin and induces diarrhea by activating the phospholipase C–inositol trisphosphate pathway upon release from infected cells, resulting in Ca^2+^ efflux from the endoplasmic reticulum and enhanced Cl^−^ secretion via Ca^2+^-dependent Cl^−^ channels (40). In addition to its enterotoxic activity, the NSP4_114–135 peptide is a fully non-competitive inhibitor of SGLT1 (14). Inhibition of SGLT1 has been reported to block *C. parvum* infection by preventing microvillus expansion required for parasite invasion (41). Consistent with this mechanism, no significant reduction in *C. parvum* infection was observed at 3 h post-infection, when the parasite is primarily in the adhesion and early invasion stage. These findings are consistent with the possibility that the NSP4 peptide interferes with later stages of host cell invasion rather than initial attachment. In this study, we focused on NSP4 because it is the only rotavirus protein known to directly alter epithelial transport and barrier function. Although other rotaviral proteins may indirectly influence *C. parvum* infection through modulation of host immune responses, direct interference with host cell transporters is most plausibly mediated by NSP4. Future studies will be required to directly test this hypothesis *in vivo*, for example by measuring NSP4 levels and SGLT1 activity in intestinal tissues from mono-infected and co-infected calves, or by experimentally manipulating rotavirus infection prior to *C. parvum* challenge.

In co-infection experiments *in vitro* using human intestinal epithelial cells and *in vivo* in mice, co-infection with human rotavirus WI61 strain and *C. parvum* resulted in a decreased number of rotavirus-infected cells when *C. parvum* infection preceded rotavirus infection, without affecting the severity of diarrhea (42). The authors attributed this to the induction of host antiviral immune responses by *C. parvum*-associated dsRNA virus (*Cryptosporidium parvum* virus 1, CSpV1). In Japan, CSpV1 was detected in both *C. parvum* isolates from calves and the experimentally used HNJ-1 strain used in this study (43). This observation highlights that *C. parvum* itself can harbor viral elements, supporting the broader concept that virus–parasite interactions may influence parasite biology and pathogenicity, as suggested by our findings for BRV–*C. parvum* co-infection. These findings indicate that co-infection with BRV and *C. parvum* involves complex interactions that are influenced by the sequence of infection and the presence of persistent *C. parvum* infections.

Cryptosporidiosis is a substantial issue in calves, as even a few oocysts (500 oocysts) can establish infection (44). In endemic farms, nearly 100% of calves develop cryptosporidiosis; however, clinical symptoms due to *C. parvum* are typically limited to the pre-weaning period. Therefore, mitigating the severity of cryptosporidiosis during this period is crucial for improving cattle productivity. Our findings indicate that BRV–*C. parvum* co-infection is associated with a shortened duration of diarrhea in calves and that BRV NSP4 inhibits *C. parvum* infection *in vitro*, raising the possibility that viral-parasite interactions could be exploited for future control strategies for cryptosporidiosis though a direct *in vivo* role of NSP4 remains to be demonstrated. In farms where BRV is endemic, maternal vaccination against BRV or adequate colostrum administration may contribute to the control of *C. parvum*. Furthermore, approaches that modulate host responses through viral components, such as NSP4 peptides or viral nucleic acids, warrant further investigation as potential measures against *C. parvum* infection. In this study, we acknowledge the limitation of the small number of calves examined, particularly the low number of animals showing natural co-infection. Conducting controlled infection experiments in calves is ethically and logistically challenging, and our analysis was therefore based on naturally occurring infections. In such field-based settings, it is not feasible to manipulate infection timing or repeat sampling, as some animals may not develop co-infection or may be sold before longitudinal data can be obtained. These *in vitro* analyses provided complementary evidence that supports the field observations and strengthens the overall interpretation of rotavirus-induced modulation of *C. parvum* infection. The limited number of calves examined may reduce the statistical robustness of our findings; however, the consistent trends observed in both field and cell-culture experiments support the validity of our conclusions. Recently, it has been reported that the intestinal fungal community of calves influences the infectivity of *C. parvum* (45).

These findings suggest that interactions among multiple microorganisms can regulate pathogenic infections, providing important insights for the development of future infection control strategies. Future studies should further elucidate the mechanisms underlying BRV and *C. parvum* co-infection in cattle.

## Data Availability

The original contributions presented in the study are included in the article/Supplementary material, further inquiries can be directed to the corresponding author.
